# Evaluation of neuroimaging findings in thalamic lesions: what can we think?

**DOI:** 10.1590/0100-3984.2020.0129

**Published:** 2021

**Authors:** Bruno Niemeyer de Freitas Ribeiro, Edson Marchiori

**Affiliations:** 1 Department of Radiology, Instituto Estadual do Cérebro Paulo Niemeyer, Rio de Janeiro, RJ, Brazil.; 2 Department of Radiology, Hospital Casa de Portugal / 3D Diagnóstico por Imagem, Rio de Janeiro, RJ, Brazil.; 3 Universidade Federal do Rio de Janeiro (UFRJ), Rio de Janeiro, RJ, Brazil.

**Keywords:** Thalamus, Magnetic resonance imaging, Tomography, X-ray computed, Demyelinating diseases, Infections, Tálamo, Ressonância magnética, Tomografia computadorizada, Doenças desmielinizantes, Infecções

## Abstract

The diseases that affect the thalamus are heterogeneous in their etiologies, including infectious, inflammatory, vascular, toxic-metabolic, and neoplastic causes. It is often difficult to make the clinical differentiation between different entities. Within this context, computed tomography and magnetic resonance imaging have come to be of fundamental importance for defining the etiology and planning the treatment. In this pictorial essay, we will illustrate the main causes of diseases affecting the thalamus, discussing the possible differential diagnoses, as well as the most relevant imaging aspects.

## Abstract (pt)

As doenças que envolvem os tálamos incluem um grupo heterogêneo, englobando causas infecciosas, inflamatórias, vasculares, tóxico-metabólicas e neoplásicas. Muitas vezes a diferenciação clínica entre as diversas entidades é difícil, e dentro desse contexto, a tomografia computadorizada e a ressonância magnética assumiram fundamental importância na busca da definição etiológica e para guiar a conduta terapêutica. Neste ensaio iconográfico ilustraremos as principais causas de acometimento talâmico, discutindo seus possíveis diagnósticos diferenciais, bem como seus aspectos de imagem mais relevantes.

## INTRODUCTION

The thalamus is a structure composed of gray matter, located on either side of the third ventricle and comprising multiple nuclei with different functions, such as controlling consciousness, behavior, sleep, and alertness. There are numerous etiologies associated with thalamic lesions, and it is essential to investigate the clinical and biochemical history of the patient before requesting an imaging examination. Recent studies in the radiology literature of Brazil have highlighted the importance of imaging examinations for improving the diagnosis of neurological diseases^(1-5)^.

In this study, we will discuss the imaging findings of thalamic lesions on computed tomography (CT) and magnetic resonance imaging (MRI), organized by etiology, including infectious/inflammatory, vascular, toxic-metabolic, and neoplastic causes.

## INFECTIOUS AND INFLAMMATORY CAUSES

### Creutzfeldt-Jakob disease

**Figure 1.**
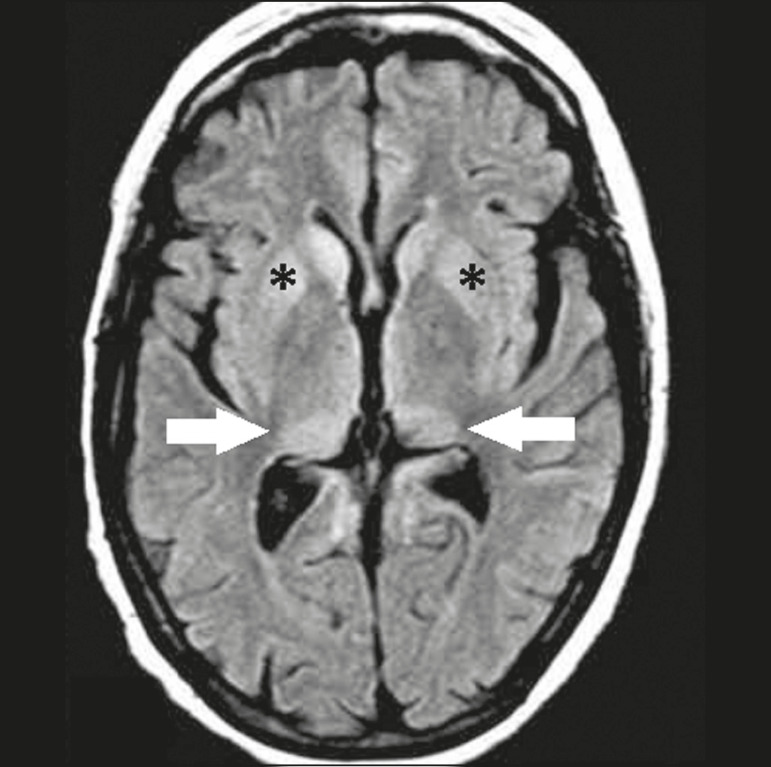
Creutzfeldt–Jakob disease in a patient with rapidly progressive cognitive decline. Axial FLAIR MRI sequence showing bilateral areas of hyperintensity in the thalamus (arrows), as well as in the caudate and lentiform nuclei (asterisks).

Creutzfeldt-Jakob disease is a rare, rapidly progressive neurodegenerative disease, with no predilection for sex, preferentially affecting individuals between the fifth and eighth decades of life. The most common form, seen in 85% of cases, is the sporadic form, although there are also familial, iatrogenic, and variant forms^(6)^. The characteristic clinical finding is a rapid decline in cognitive function. The “pulvinar” and “hockey stick” signs are typical of the variant form and are characterized by restricted diffusion on diffusion-weighted functional MRI (DfMRI), with hyperintense signals in T2-weighted and fluid-attenuated inversion recovery (FLAIR) sequences in the posterior thalamus and posteromedial thalamus, respectively (Figure 1).

### Neurotoxoplasmosis

**Figure 2.**
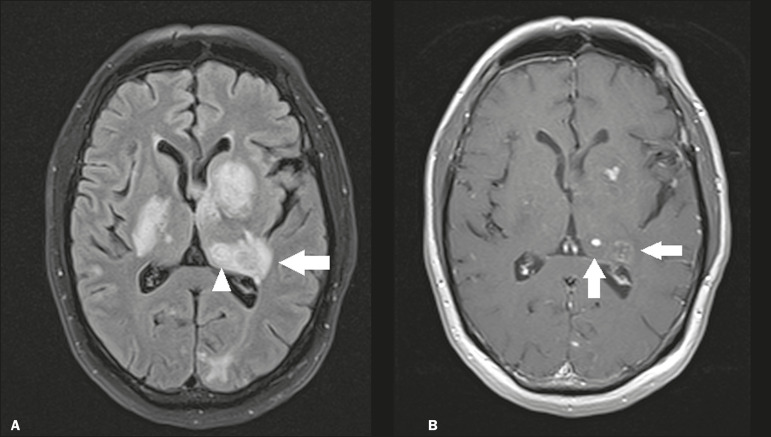
Neurotoxoplasmosis in a patient with acquired immunodeficiency syndrome who had a CD4 cell count of 126 cells/mm3. **A:** Axial FLAIR MRI sequence showing multiple hyperintense lesions in the nucleocapsular regions and in the left thalamus (arrow), with the eccentric target sign in the most medial lesion in the left thalamus (arrowhead). **B:** Contrast-enhanced axial T1-weighted MRI sequence showing nodular peripheral enhancement of the thalamic lesions (arrows).

Caused by *Toxoplasma gondii*, neurotoxoplasmosis typically occurs in immunocompromised patients^(7)^. It commonly manifests as multiple lesions affecting the nucleocapsular and thalamic regions, as well as the cortico-subcortical junction. On CT, the lesions are hypodense, with contrast uptake, and calcifications are common in treated cases. On MRI, the lesions show variable signal intensity in T2-weighted sequences and may present concentric zones of hypointense, isointense, or hyperintense signal intensity (concentric halo sign), together with contrast uptake, which can be nodular or peripheral, the latter potentially presenting the eccentric target sign (Figure 2), a common, although not pathognomonic, finding. Perilesional edema is also a common finding.

### Viral encephalitis

Numerous viruses can cause encephalitis, and thalamic involvement is quite common in flavivirus infection. MRI can demonstrate symmetrical or asymmetric thalamic involvement, characterized by hyperintensity in T2-weighted sequences, together with enhancement and variable restricted diffusion on DfMRI. There can be foci of bleeding, even in cases of dengue encephalitis^(8)^.

### Acute disseminated encephalomyelitis

**Figure 3.**
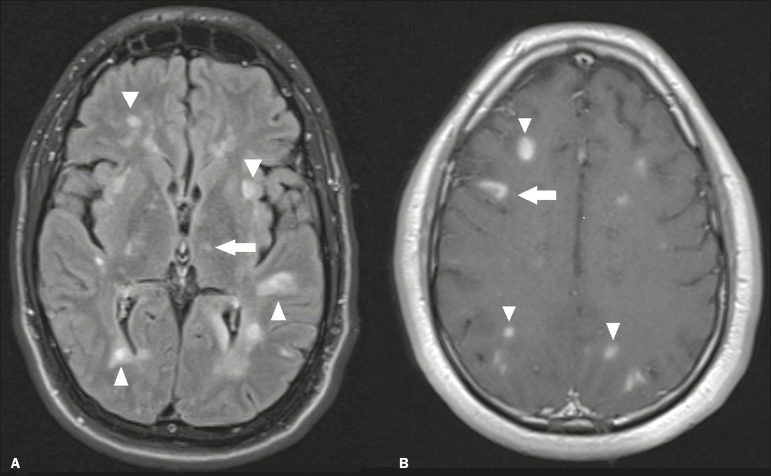
Acute disseminated encephalomyelitis after viral infection. **A:** Axial FLAIR MRI sequence showing multiple hyperintense lesions throughout the white matter on both sides (arrowheads) and slightly affecting the left thalamus (arrow). **B:** Contrast-enhanced axial T1-weighted MRI sequence, acquired at a higher level, showing nodular enhancement of some lesions (arrowheads) and discontinuous annular enhancement (arrow) characteristic of demyelinating lesions.

Acute disseminated encephalomyelitis is a demyelinating, immune-mediated disease that is classically monophasic. It is most common in children and is usually preceded by viral infection or vaccination. On MRI, it presents as small or swollen, asymmetric lesions that are hyperintense in T2-weighted sequences, with variable contrast enhancement, which may have a peripheral and discontinuous appearance, thalamic involvement (Figure 3) being seen in approximately 40% of cases^(6)^. It can be accompanied by a longitudinally extensive spinal cord lesion. Acute necrotizing encephalopathy in childhood is another immune-mediated disease that goes into the differential diagnosis of bilateral thalamic involvement in pediatric patients.

## VASCULAR CAUSES

### Deep cerebral venous thrombosis

**Figure 4.**
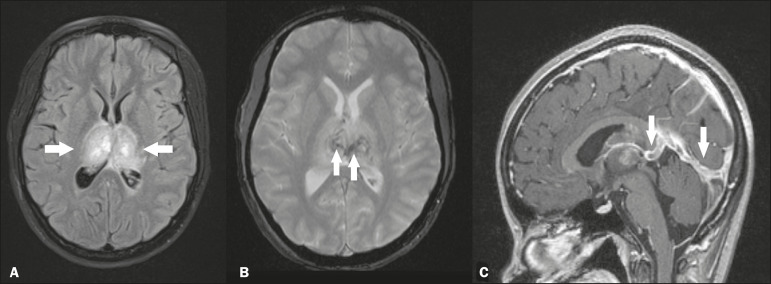
Deep cerebral venous thrombosis in a female patient who used oral contraceptives. A: Axial FLAIR MRI sequence showing hyperintensity and a volume increase in the thalamus (arrows). B: Axial T2*-weighted MRI sequence showing hypointense foci within thalamic lesions (arrows), secondary to hemoglobin degradation products. C: Sagittal-enhanced axial T1-weighted MRI sequence showing a filling defect affecting the straight sinus (arrows). Source: Courtesy of Dr. Márcio Tadeu Vieira de Brito.

Deep cerebral venous thrombosis can occur at any age, with a predilection for the female sex, being more common in women using oral contraceptives, as well as in women who are pregnant or have recently given birth. The clinical presentation is variable, although the evolution is more rapid than in cases of superficial venous thrombosis. When it affects the internal cerebral veins and the straight sinus, it usually causes a volume increase in the thalamus, due to edema, which appears on MRI as an area of hyperintensity in T2-weighted and FLAIR sequences (Figures 4A and 4B), potentially evolving to restricted diffusion on DfMRI and hypointense foci in susceptibility-weighted sequences, due to hemorrhage. On CT angiography and magnetic resonance angiography, a filling defect can be seen, involving the straight sinus, the internal cerebral veins, or both (Figure 4C).

### Thalamic infarcts

**Figure 5.**
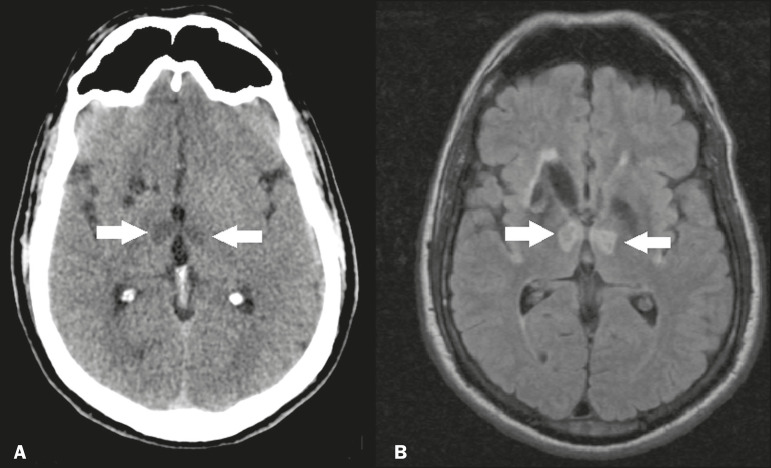
Bilateral thalamic infarcts in a patient with a sudden decrease in the level of consciousness. **A:** Axial CT slice showing areas of hypodensity in the thalamus (arrows). **B:** Axial FLAIR MRI sequence showing areas of hyperintensity in the thalamus (arrows).

The arterial supply of the thalamus is highly variable, its anteroinferior aspect typically receiving its blood supply from the perforating arteries of the anterior circulation, the remainder receiving its blood supply from the posterior circulation, mainly from branches of the P1 and P2 segments of the posterior cerebral artery^(9)^. Thalamic infarcts can give rise to numerous clinical syndromes, depending on the affected region, the most common site being the inferolateral region, the involvement of which can manifest as severe pain not relieved by analgesics, sensory loss, and ataxic hemiparesis^(9)^. In acute cases, the CT findings can be normal, later evolving to areas of hypodensity in the affected region (Figure 5A). MRI is more sensitive, DfMRI showing early restricted diffusion in the thalamus, with variable involvement of the midbrain (Figure 5B).

### Posterior reversible encephalopathy syndrome

Posterior reversible encephalopathy syndrome is an entity of varying etiology. Its pathophysiology is characterized by endothelial injury and dysfunction in the mechanisms of cerebral autoregulation. The clinical picture is acute/subacute, classically characterized by headache, a decreased level of consciousness, visual disturbances, and seizures. The typical MRI finding is bilateral symmetrical hyperintensity in T2-weighted and FLAIR sequences, affecting the cortex and the subcortical region, with a predilection for the parieto-occipital region. Involvement of the thalamus is rare, being most common in the central variant, as is involvement of the cerebellum, brain stem, and nucleocapsular region^(10)^.

### Hypoxic-ischemic encephalopathy

**Figure 6.**
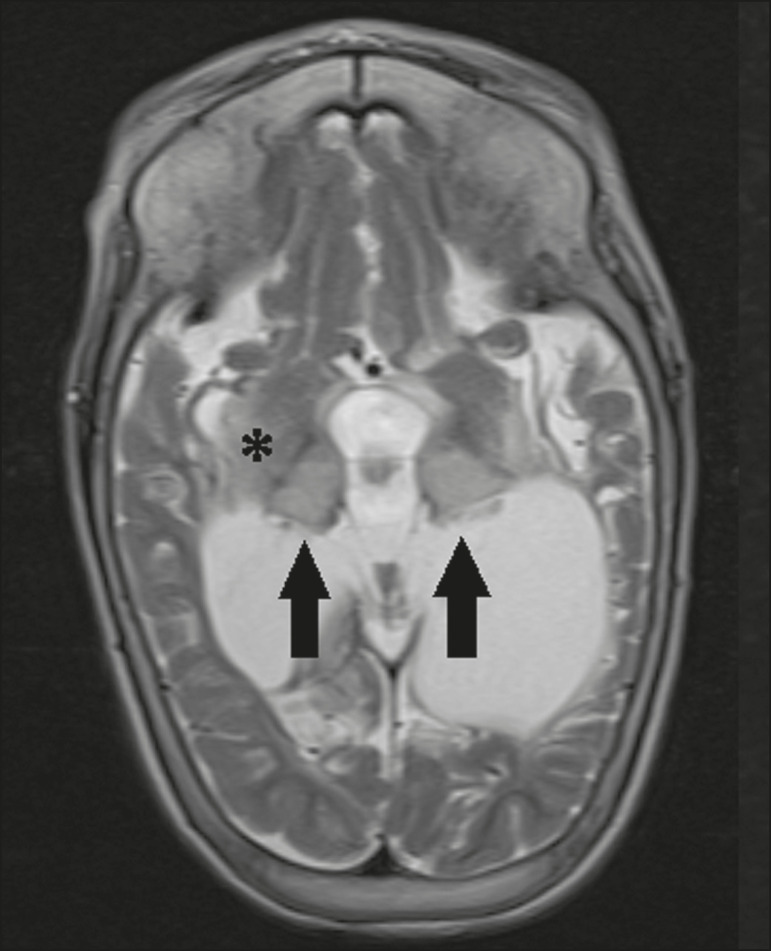
Severe hypoxic-ischemic encephalopathy. Axial T2-weighted MRI sequence showing areas of hypodensity, together with volume reductions, in the thalamus (arrows) and the nucleocapsular region (asterisk). Note also the pronounced diffuse thinning of the white matter.

Hypoxic-ischemic encephalopathy can occur at any point in life. In infants, neuroimaging presents different aspects, depending on the severity, the duration of hypoxia, and the age of the child. Thalamic involvement is common in cases of severe hypoxia and in infants at 32-36 weeks of age, typically being accompanied by nucleocapsular involvement and a reduction in white matter volume (Figure 6), probably due to changes in the thalamocortical projections^(11)^. In adults, most cases manifest as cardiopulmonary arrest, and CT may show diffuse cerebral edema with loss of differentiation between the white and gray matter, together with reversal of the normal attenuation of the white/gray matter and white cerebellum, the latter two having a worse prognosis. MRI is the most sensitive method, the DfMRI sequence showing changes in the first hours^(11)^.

## TOXIC-METABOLIC CAUSES

### Wernicke’s encephalopathy

**Figure 7.**
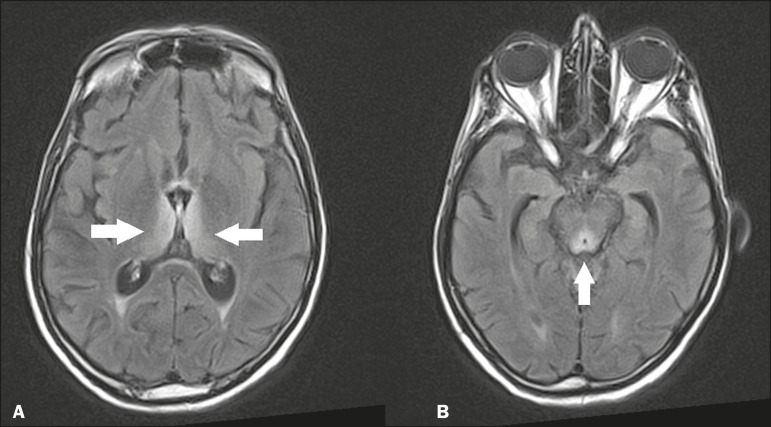
Wernicke’s encephalopathy in a malnourished alcoholic patient. **A:** Axial FLAIR MRI sequence showing symmetric areas of hyperintensity in the paraventricular regions of the thalamus (arrows). **B:** Axial FLAIR MRI sequence showing hyperintensity in the periaqueductal region (arrow).

Wernicke’s encephalopathy is an acute neuropsychiatric condition that manifests as ophthalmoplegia/nystagmus, ataxia, and mental confusion secondary to thiamine deficiency, classically associated with alcoholism and malnutrition. On MRI, the typical findings are symmetric areas of hyperintensity in T2-weighted and FLAIR sequences in the paraventricular regions of the thalamus, mammillary bodies, hypothalamus, periaqueductal region, and floor of the fourth ventricle (Figure 7). There can be contrast enhancement and restricted diffusion^(6)^.

### Status epilepticus

**Figure 8.**
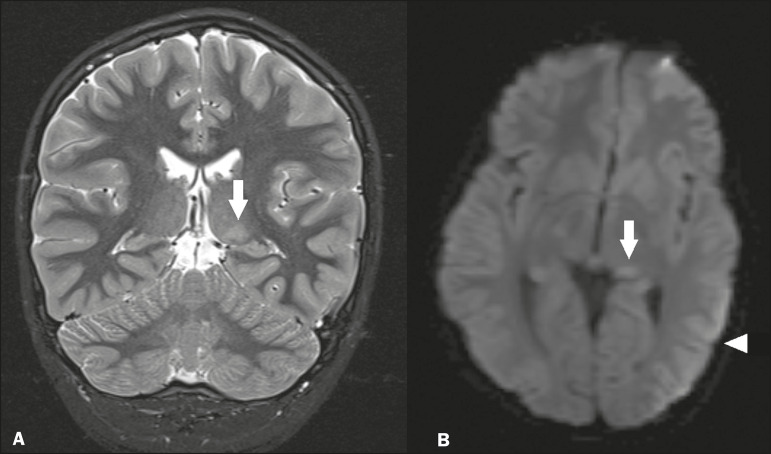
Status epilepticus secondary to eclampsia. A: Coronal short-tau inversion recovery MRI sequence showing hyperintensity in the left thalamus (arrow). B: Axial DfMRI sequence showing hyperintensity in the pulvinar region of the left thalamus (arrow), as well as in the cortices of the ipsilateral temporal and occipital lobes (arrowhead).

Status epilepticus is characterized by prolonged or recurrent seizures, lasting approximately 30 min. In up to 50% of cases, it occurs in patients without a previous history of seizures; it may be related to numerous factors, including hypoxia, encephalitis, and eclampsia^(12)^. The most common alteration on MRI is hyperintensity in T2-weighted and FLAIR sequences affecting the cortical gray matter or the subcortical white matter, which may be accompanied by contrast enhancement and restricted diffusion on DfMRI. There can also be involvement at other sites, such as the hippocampus and thalamus, the latter typically being affected in its pulvinar region (Figure 8).

### Other toxic-metabolic and degenerative causes

**Figure 9.**
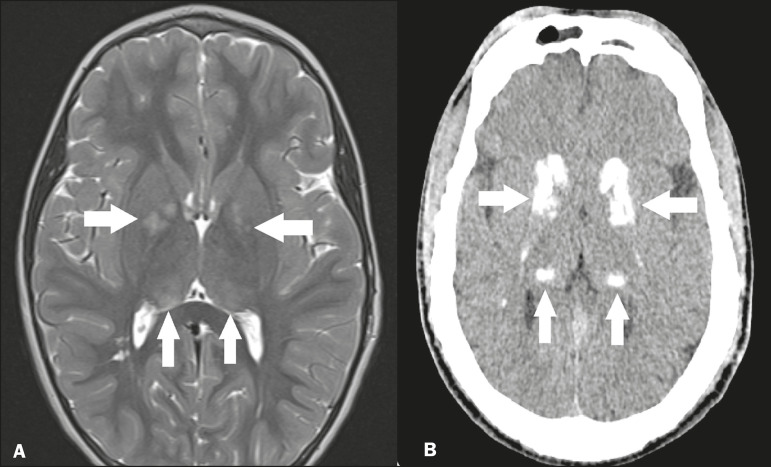
Other toxic-metabolic and degenerative causes of thalamic lesions. **A:** Axial T2-weighted MRI sequence showing small hyperintense foci in the thalamus and nucleocapsular regions (arrows) in a six-year-old patient with neurofibromatosis type 1 (image courtesy of Dr. Márcio Tadeu Vieira de Brito). **B:** Axial CT slice showing calcifications in the thalamus and nucleocapsular regions (arrows) in a patient with Fahr’s disease who presented with progressive psychosis.

Other diseases that can affect the thalamus include osmotic demyelination syndrome, Fahr’s disease, mitochondrial encephalopathy, maple syrup urine disease, gangliosidosis, and neurofibromatosis type 1 (Figure 9). In patients with such diseases, the accompanying findings can guide the diagnosis^(6,13-15)^: involvement of the pons, with preservation of its periphery and corticospinal tracts, is indicative of osmotic demyelination syndrome; subcortical calcifications and dentate nuclei are indicative of Fahr’s disease; infarct-like lesions, together with a lactate peak on proton magnetic resonance spectroscopy, are indicative of mitochondrial encephalopathy; restricted diffusion on DfMRI in the thalamus, brainstem, and cerebellum is indicative of maple syrup urine disease; and a hyperdense thalamus on CT is indicative of gangliosidosis.

## NEOPLASTIC CAUSES

### Gliomas

**Figure 10.**
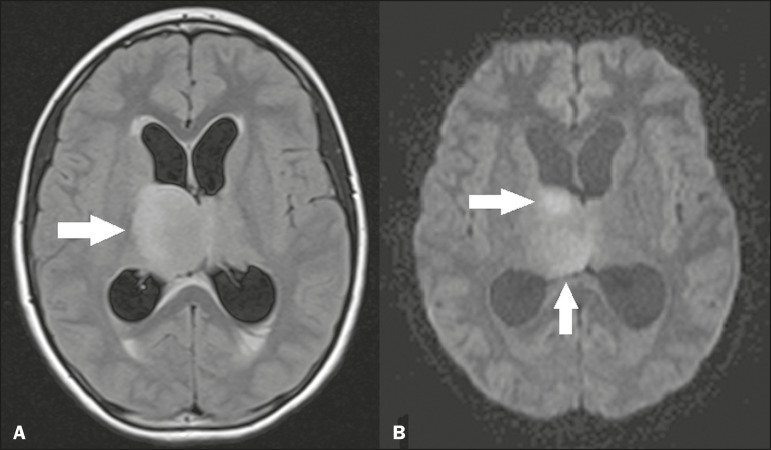
Histopathologically proven diffuse midline glioma with a histone H3-K27M mutation. **A:** Axial FLAIR MRI sequence showing an expansile hyperintense lesion in the right thalamus (arrow). **B:** Axial DfMRI sequence demonstrating hyperintense foci (arrows) within the lesion shown in **A**.

Many tumors can affect the thalamus. The main primary neoplasm involving the thalamus is diffuse midline glioma with a histone H3-K27M mutation, typically being characterized on MRI as hyperintense lesions in T2-weighted and FLAIR sequences, with restricted diffusion on DfMRI and variable contrast enhancement on gadolinium contrast-enhanced images (Figure 10).

### Metastases

**Figure 11.**
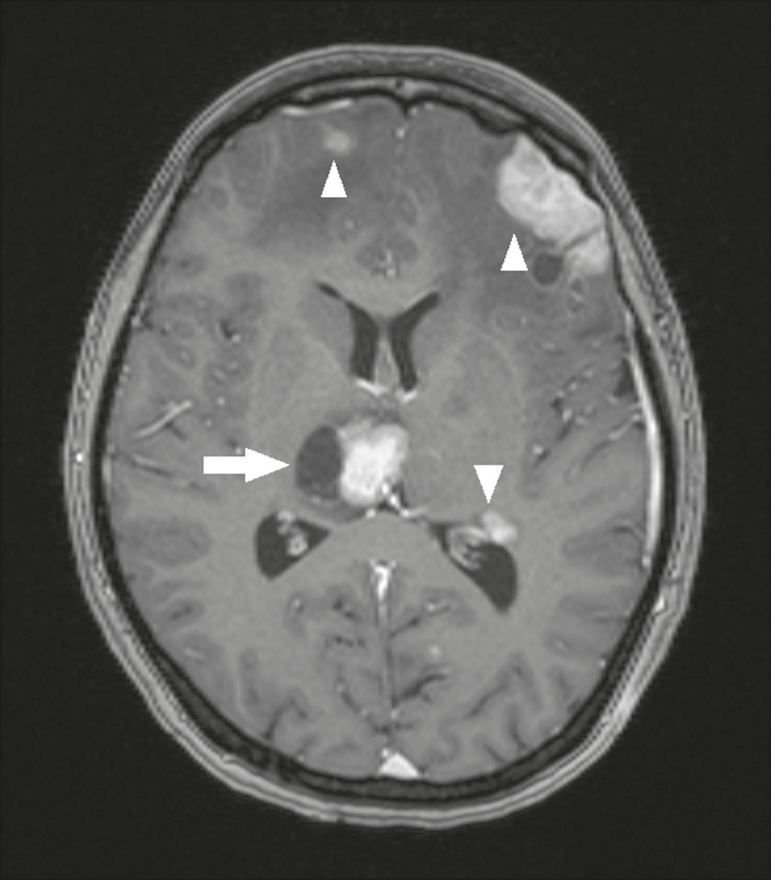
Metastases in a patient with clear cell renal cell carcinoma. Contrast- enhanced axial T1-weighted MRI sequence showing an expansile lesion, with contrast uptake, in the right thalamus, as well as other multiple lesions on both sides (arrowheads).

Metastases constitute the main malignant condition affecting the brain, being most common at the cortico-subcortical junction, due to the greater vascularization in that region, and less common in the thalamus. On CT and MRI, metastases are characterized by heterogeneous lesions with contrast uptake and signs of hyperperfusion (Figure 11). They should be considered in patients with known primary neoplasia or when multiple brain lesions are observed.

## CONCLUSION

Imaging is a very useful tool in patients with thalamic involvement and can add fundamental information for the diagnosis, treatment planning, and follow-up of such involvement. Radiologists should be aware of the neuroimaging patterns in order to be able to contribute to the clinical decision-making process.
